# The tumour suppressor Ras-association domain family protein 1A (RASSF1A) regulates TNF-α signalling in cardiomyocytes

**DOI:** 10.1093/cvr/cvu111

**Published:** 2014-04-28

**Authors:** Tamer M.A. Mohamed, Min Zi, Sukhpal Prehar, Arfa Maqsood, Riham Abou-Leisa, Loan Nguyen, Gerd P. Pfeifer, Elizabeth J. Cartwright, Ludwig Neyses, Delvac Oceandy

**Affiliations:** 1 Institute of Cardiovascular Sciences, University of Manchester, Oxford Road, Manchester M13 9PT, UK; 2 Faculty of Pharmacy, Zagazig University, EL-Sharkiah, Egypt; 3 J David Gladstone Research Institutes, San Francisco, CA, USA; 4 Division of Biology, Beckman Research Institute of the City of Hope, Duarte, CA, USA

**Keywords:** Calcium transient, Contractile function, RASSF1A, Signal transduction, Tumour necrosis factor alpha

## Abstract

**Aims:**

Tumour necrosis factor-α (TNF-α) plays a key role in the regulation of cardiac contractility. Although cardiomyocytes are known to express the TNF-α receptors (TNFRs), the mechanism of TNF-α signal transmission is incompletely understood. The aim of this study was to investigate whether the tumour suppressor Ras-association domain family protein 1 isoform A (RASSF1A) modulates TNF-α signalling in cardiomyocytes.

**Methods and results:**

We used RASSF1A knockout (RASSF1A^−/−^) mice and wild-type (WT) littermates in this study. Acute stimulation with a low dose of TNF-α (10 µg/kg iv) increased cardiac contractility and intracellular calcium transients' amplitude in WT mice. In contrast, RASSF1A^−/−^ mice showed a blunted contractile response. Mechanistically, RASSF1A was essential in the formation of the TNFR complex (TNFRC), where it functions as an adaptor molecule to facilitate the recruitment of TNFR type 1-associated death domain protein and TNFR-associated factor 2 to form the TNF-α receptor complex. In the absence of RASSF1A, signal transmission from the TNF-α receptor complex to the downstream effectors, such as cytoplasmic phospholipase A2 and protein kinase A, was attenuated leading to the reduction in the activation of calcium handling molecules, such as L-type Ca^2+^ channel and ryanodine receptors.

**Conclusion:**

Our data indicate an essential role of RASSF1A in regulating TNF-α signalling in cardiomyocytes, with RASSF1A being key in the formation of the TNFRC and in signal transmission to the downstream targets.

## 1. Introduction

Tumour necrosis factor-α (TNF-α) is a pro-inflammatory cytokine that plays an important role in the heart, in particular, in the regulation of contractility and left ventricular (LV) remodelling. Mice with cardiomyocyte-specific overexpression of TNF-α developed dilated cardiomyopathy, altered cardiac contractility, and abnormal intracellular calcium dynamics.^1–4^ TNF-α effects in the heart seem to be dose- and time-dependent. For example, treatment of adult cardiomyocytes with TNF-α for 12 h protects against hypoxic injury.^5^ Also, low dose of TNF-α treatment induces hypertrophic growth in isolated myocytes,^6^ whereas acute treatment with a low dose of TNF-α produces a positive inotropic effect in conscious dogs.^7^ Moreover, TNF-α appears to produce biphasic effects on cardiac contractility: at low concentrations, TNF-α increases the amplitude of [Ca^2+^]_i_ transients and contraction, whereas at high concentrations TNF-α impairs electrically stimulated [Ca^2+^]_i_ transients and contraction.^8,9^

TNF-α is involved in a number of pathological conditions. For example, TNF-α is one of the key mediators of systemic endotoxemia.^10^ Serum TNF-α level is dramatically elevated in endotoxemia and may be important in determining cardiac contractile function in this condition.

TNF-α mainly exerts its biological effects through binding to two different types of TNF-α receptors, TNFR1 and TNFR2, both of which are expressed in cardiomyocytes.^11^ Upon binding to TNF-α, the TNFRs recruit a number of molecules to form the TNFR complex (TNFRC), which in turn initiates the activation of the downstream signalling cascade.^12^ Here, we discover for the first time that a Ras effector/interacting protein, the Ras-association domain family protein 1 isoform A (RASSF1A), is essential for the formation of the TNFRC in cardiomyocytes. RASSF1A is a tumour suppressor molecule that in non-cardiomyocytes regulates a number of important cellular processes such as apoptosis, cell growth and viability, and also the cell cycle. RASSF1A lacks enzymatic activity and it exerts its functions mainly via interaction with and modulation of other molecules.^13,14^ RASSF1A is expressed in the heart, but its involvement in the regulation of TNF-α signalling in cardiomyocytes is unknown.

In the present study, we used a RASSF1A knockout mouse model, as well as isolated cardiomyocytes with genetic ablation or overexpression of RASSF1A, to demonstrate the pivotal role of this molecule in mediating the TNF-α-induced contractile response in cardiomyocytes and in the whole heart.

## 2. Methods

### 2.1 Plasmids

The human RASSF1A cDNA was a gift of Dr Geoffrey Clark (Louisville, KY, USA). The generation of RASSF1A deletion mutants has been described previously.^15^ Plasmid containing human TNFR-associated factor 2 (TRAF2) cDNA was a gift of Dr John Kyriakis (Boston, MA, USA; Addgene plasmid #21586).^16^ Plasmids containing human TNFR1 and human TNFR type 1-associated death domain protein (TRADD) were obtained from Origene. For the NFκB activity assay, we used a luciferase construct containing four tandem repeats of NFκB-binding sites (Clontech). For RASSF1A gene silencing, we used shRNA targeting rat RASSF1A driven by the U6 promoter and shRNA containing scrambled sequence driven by the same promoter was used as the control.

### 2.2 Generation of adenoviral constructs

Adenoviruses were generated by cloning cDNAs to the pAd/CMV/V5-DEST vector (Invitrogen) using the Gateway system following the manufacturer's recommended methods. pENTR11 vector (Invitrogen) was used as the shuttle system.

### 2.3 Animals

We used mice with systemic genetic ablation of the *Rassf1a* gene as described previously.^17^ All animal experiments were performed on 16- to 20-week-old mice in accordance with the UK Animals (Scientific Procedures) Act 1986 and were approved by the University of Manchester Ethics Committee.

### 2.4 Haemodynamic analysis

*In vivo* haemodynamic analyses were performed as described previously.^18^ Briefly, mice were anaesthetized by intraperitoneal injection of tribromoethanol [240 mg/kg body weight (BW)] and placed on a heat pad at 37°C. A 1.4-Fr pressure–volume catheter (Millar Instruments) was inserted into the left ventricle via the right carotid artery. Pressure–volume signals were recorded first under basal conditions and then recorded 30 min after intravenous injection of TNF-α (10 µg/kg BW).

### 2.5 Isolation of mouse adult cardiomyocytes and neonatal rat cardiomyocytes

Adult cardiomyocytes were isolated from 3- to 4-month-old wild-type (WT) or RASSF1A^−/−^ mice, using methods described previously.^18^ Neonatal rat cardiomyocytes were isolated from 1- to 3-day-old Sprague-Dawley rats. Details of the isolation methods are provided in Supplementary material online, Methods.

### 2.6 Intracellular calcium transient measurements

Isolated adult cardiomyocytes were loaded with calcium ratiometric fluorescent dye (Indo-l). In order to measure the cytosolic calcium, the myocytes were perfused with Tyrode solution and then field stimulated at a frequency of 1 Hz. Calcium changes during myocyte contraction were recorded before and after stimulation with either TNF-α (10 ng/mL) or isoproterenol (100 nM) as previously described.^18^ To assess the involvement of cytoplasmic phospholipase A2 (cPLA2), we treated cardiomyocytes with cPLA2 inhibitor AACOCF3 (Calbiochem) at a dose of 20 μM or cPLA2 activator peptide PLAP (Santa Cruz Biotechnology) at 1 μM. Details of calcium transient measurement are provided in Supplementary material online, Methods.

### 2.7 Data analysis

Data are presented as mean ± SEM. Statistical analyses were carried out using the Student's *t*-test, one- or two-way analysis of variance (ANOVA), where appropriate. Values were considered significant if *P* < 0.05 [see Supplementary material online, Methods for western blot, immunoprecipitation, cPLA2, PKA, calcium–calmodulin-dependent kinase II (CaMKII), and NFκB activity assays].

## 3. Results

### 3.1 RASSF1A^−/−^ mice showed a blunted contractile response following acute treatment with a low dose of TNF-α

**Figure 1:**
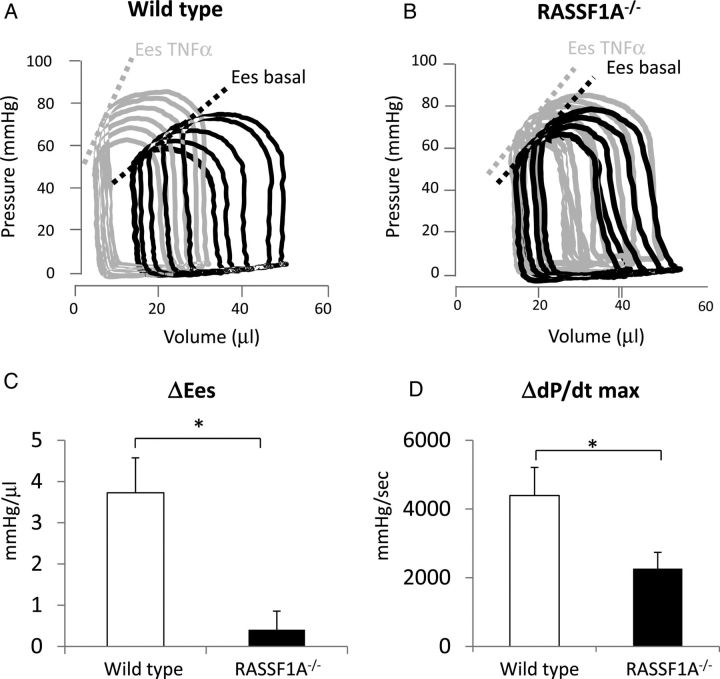
Effect of acute TNF-α stimulation on cardiac contractility in RASSF1A^−/−^ and WT littermates. Representative pressure–volume loops from (*A*) WT and (*B*) RASSF1A^−/−^ mice under basal conditions (blue line) and 30 min after intravenous injection with TNF-α (10 µg/kg BW) (red line). Dotted lines represent end-systolic elastance (Ees). (*C*) The change in end-systolic elastance (ΔEes) after TNF-α injection was measured. WT mice showed a significantly greater increase in Ees in response to TNF-α stimulation. (*D*) The change in the maximum rate of developed pressure (Δd*P*/d*t* max) was also higher in WT mice (*n* = 7–10, *P < 0.05).

To assess the involvement of RASSF1A in TNF-α signalling, we injected a low dose of TNF-α (10 µg/kg BW) intravenously in WT and RASSF1A^−/−^ mice. We analysed pressure–volume loops to assess indices of contractility (*Figure 1A* and *B*, and *Table 1*). There was no significant difference in basal haemodynamic indices between WT and RASSF1A^−/−^ mice. However, paired analysis between basal and 30-min post-TNF-α infusion showed that acute TNF-α treatment significantly increased end-systolic LV pressure and decreased end-diastolic LV pressure without creating any changes in heart rate and other diastolic parameters in both WT and knockout mice (*Table 1*). The changes in indices of contractility (Δd*P*/d*t*_max_ and ΔEes [end-systolic elastance]) in response to TNF-α were dramatically different between WT and RASSF1A^−/−^ mice. Both Ees and d*P*/d*t*_max_ were significantly enhanced in WT animals (*Figure 1C* and *D*), indicating that acute treatment with a low dose of TNF-α increased cardiac contractility, which was consistent with previously published data.^7–9^ Interestingly, RASSF1A^−/−^ mice showed a blunted contractile response following treatment with the same dose of TNF-α as indicated by Δd*P*/d*t*_max_ and ΔEes (*n* = 7–10, *P* < 0.05) (*Figure 1C* and *D*). These data suggested that RASSF1A is involved in modulating the cardiac contractile response following TNF-α stimulation.

**Table 1:** Haemodynamic parameters of WT and RASSF1A^−/−^ mice at basal condition and after treatment with TNF-α

		WT				RASSF1A^−/−^ (KO)			*t*-test difference after TNF-α treatment WT vs. KO
	Basal	+ TNF-α (30 min)	Difference after TNF-α treatment (Δ)	Paired *t*-test WT basal vs. WT TNF-α	Basal	+ TNF-α (30 min)	Difference after TNF-α treatment (Δ)	Paired *t*-test KO basal vs. KO TNF-α	
Heart rate (bpm)	415 ± 21	454 ± 14	39 ± 21	NS	379 ± 8	380 ± 16	1 ± 15	NS	NS
Pes (mmHg)	79 ± 3	93 ± 6	14 ± 3	*P* < 0.01	77 ± 5	84 ± 5	7 ± 3	*P* < 0.05	NS
Ped (mmHg)	6.2 ± 0.7	2.1 ± 0.5	−4.1 ± 0.8	*P* < 0.01	7.4 ± 1.1	2.0 ± 0.7	−5.4 ± 1.3	*P* < 0.01	NS
d*P*/d*t* min (mmHg/s)	−4771 ± 499	−5931 ± 508	−1160 ± 359	NS	−4213 ± 392	−4523 ± 274	−310 ± 367	NS	NS
Tau (ms)	7.1 ± 0.8	6.5 ± 0.4	−0.6 ± 0.58	NS	7.0 ± 0.4	8.1 ± 0.5	1.1 ± 0.57	NS	NS

bpm, beat per minute; Pes, end-systolic pressure; Ped, end-diastolic pressure; d*P*/d*t*, rate of ventricular pressure change; Ees, end-systolic elastance; Tau, relaxation time constant; WT, wild type.

### 3.2 RASSF1A modulates TNF-α signalling in isolated adult cardiomyocytes

**Figure 2:**
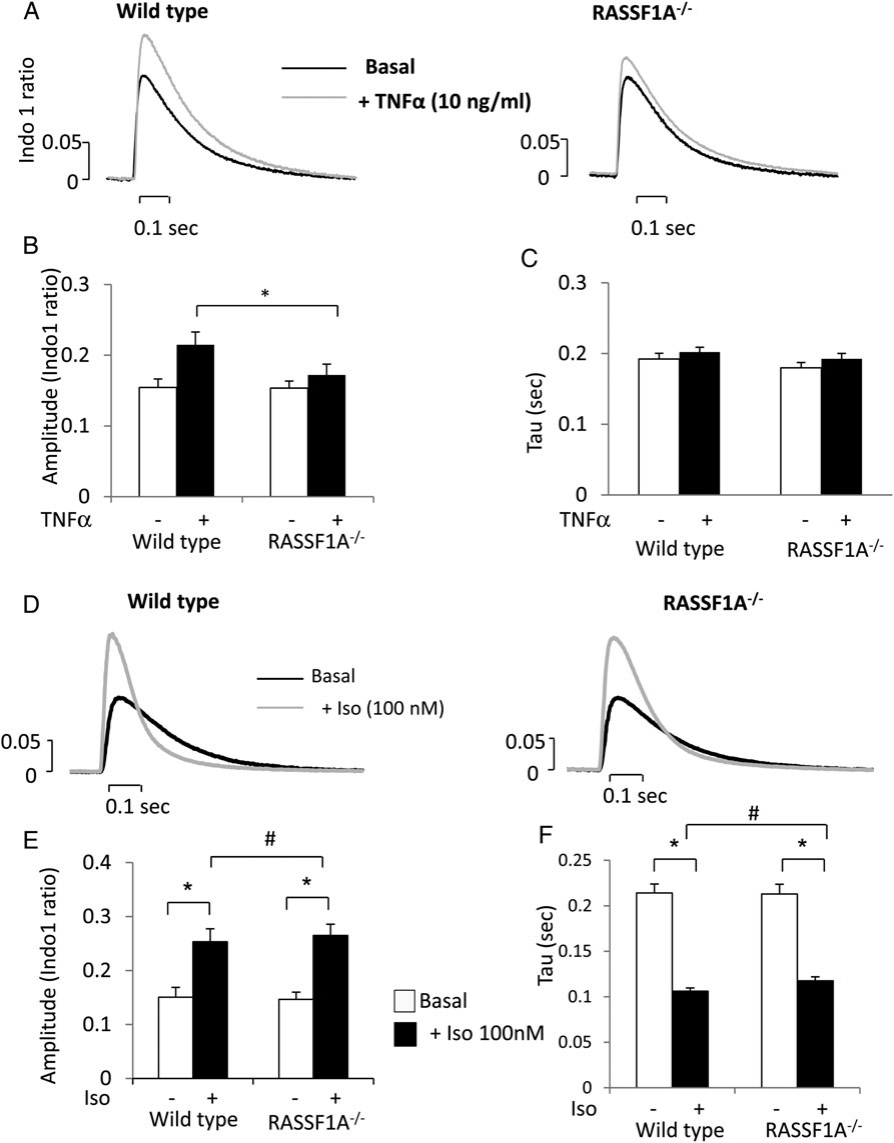
Effect of acute TNF-α or isopretorenol stimulation on calcium transients in isolated adult cardiomyocytes from RASSF1A^−/−^ mice and WT littermates. (*A*) Representative calcium transient traces from WT and RASSF1A^−/−^ cardiomyocytes loaded with Indo-1 dye before (black line) and after stimulation with TNF-α (10 ng/mL) for 30 min (red line). (*B*) Quantification of calcium transient amplitude showed that WT myocytes displayed higher amplitude following TNF-α treatment compared with RASSF1A^−/−^ cardiomyocytes. *Two-way ANOVAs showed a significant interaction between two factors tested (genotype vs. TNF-α, *n* = 22–24 myocytes from four independent animals in each group). (*C*) However, no difference in time constant of calcium decay (Tau) was observed between WT and RASSF1A^−/−^ both basally or after TNF-α stimulation. (*D*) Representative calcium transients in response to β-adrenergic agonist isoproterenol (Iso; 100 nM) in WT and RASSF1A^−/−^ cardiomyocytes. (*E*) Quantification of calcium amplitude and (*F*) Tau. ^#^Two-way ANOVAs indicated that there was significant effect of Iso treatment but there is no significant interaction between genotype and Iso response, suggesting that there is no difference in β-adrenergic response between WT and RASSF1A^−/−^ cardiomyocytes (*n* = 20–24 myocytes from four independent animals in each group). *Post hoc multiple comparison test suggested that there were significant differences before and after Iso treatment in each group.

TNF-α injection in mice could affect various cell types such as macrophages and endothelial cells, which could trigger an immune response. Therefore, to investigate whether RASSF1A mediates TNF-α-induced contractile response as a direct effect on cardiomyocytes, we assessed the effect of low-dose TNF-α treatment (10 ng/mL for 30 min) on isolated adult WT and RASSF1A^−/−^ cardiomyocytes. Consistent with the *in vivo* data, isolated adult cardiomyocytes from WT animals showed significantly higher calcium transient amplitude in response to TNF-α stimulation (*Figure 2A* and *B*). However, RASSF1A^−/−^ cardiomyocytes showed no significant change in calcium transient amplitude following similar stimulation (*Figure 2A* and *B*). No changes in the calcium decay rate were observed in both RASSF1A^−/−^ and WT mice (*Figure 2C*). These data confirm the regulatory role of RASSF1A in cardiomyocytes and imply that the *in vivo* effect described in *Figure 1* was likely due to the direct TNF-α effect on cardiomyocytes.

Next, we analysed whether RASSF1A ablation alters the β-adrenergic pathway. Data presented in *Figure 2D–F* showed that there was no difference between WT and RASSF1A^−/−^ cardiomyocytes regarding the change in calcium amplitude and calcium decay rate following β-adrenergic agonist (isoproterenol) stimulation.

### 3.3 RASSF1A ablation alters the formation of TNFRC in cardiomyocytes

**Figure 3:**
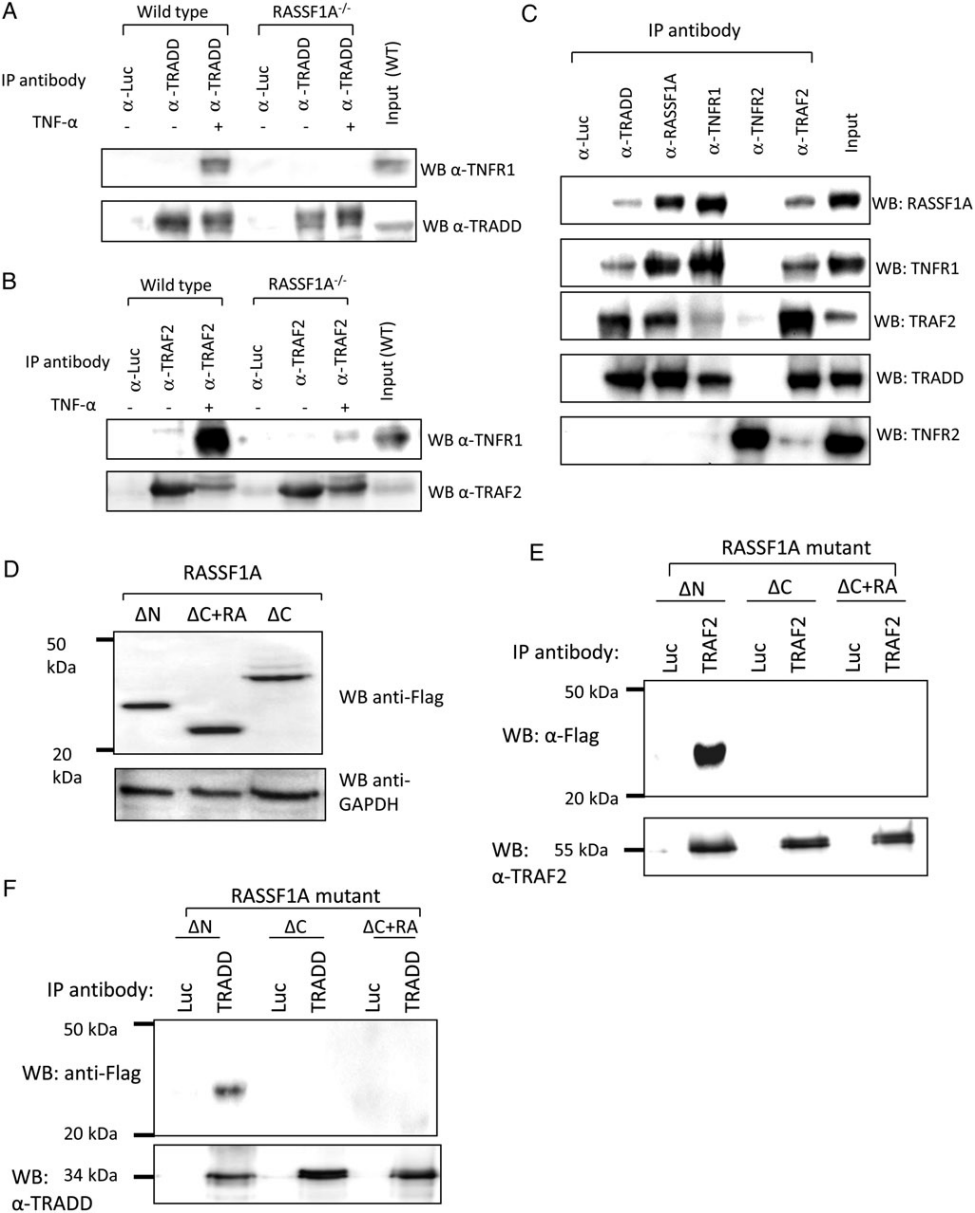
RASSF1A plays a key role in the recruitment of TRAF2 and TRADD to the TNFRC. (*A*) Isolated adult cardiomyocytes from RASSF1A^−/−^ mice and WT littermates were treated with 10 ng/mL of TNF-α for 30 min. Immunoprecipitation analysis indicated a marked reduction of TRADD and (*B*) TRAF2 co-precipitation with TNFR1 in RASSF1A^−/−^ cardiomyocytes compared with WT (*n* = 4 independent animals). (*C*) Immunoprecipitation analysis of neonatal rat cardiomyocytes (NRCMs) treated with 10 ng/mL of TNF-α. Total protein lysates were precipitated using antibodies as indicated in the figure. Western blot analyses showed that RASSF1A co-precipitated with TNFR1, TRADD, and TRAF2. However, RASSF1A did not interact with TNFR2. (*D*) Overexpression of RASSF1A deletion constructs in NRCM using adenoviral constructs. Expression was detected using an anti-Flag antibody and GAPDH as a loading control in total protein lysates. (*E*) Immunoprecipitation analysis showed that only RASSF1A-ΔN was co-precipitated with TRAF2. Both the ΔC and ΔC+RA constructs did not co-precipitate with TRAF2, suggesting that the C-terminal domain of RASSF1A was responsible for binding with TRAF2. (*F*) Similarly, the C-terminal region was also important in mediating interaction with TRADD (*n* = 3 independent experiments).

It has been described that, following activation by TNF-α, the TNFR recruits several molecules such as TRAF2 and TRADD to form a TNFRC,^19^ a process which is essential in signal transmission to the intracellular effectors. To investigate the importance of RASSF1A in the formation of TNFRC, we conducted co-immunoprecipitation experiments in adult cardiomyocytes isolated from RASSF1A^−/−^ mice and their WT littermates with or without TNF-α treatment. In WT cardiomyocytes, TNFR1 co-precipitated with both TRAF2 and TRADD and the amount of co-precipitated proteins increased following TNF-α treatment (*Figure 3A* and *B*). In contrast, there was a marked reduction in co-immunoprecipitation between TNFR1 and TRAF2/TRADD in RASSF1A^−/−^ myocytes before and after TNF-α treatment (*Figure 3A* and *B*). The levels of TNFR1, TRADD, and TRAF2 expressions were not different between WT and RASSF1A^−/−^ (see Supplementary material online, *Figure S1*). These data suggest that RASSF1A might play an essential role in the recruitment of TRADD and TRAF2 to the TNFR and the formation of TNFRC following TNF-α stimulation.

### 3.4 RASSF1A interacts with components of TNFRC

To further investigate the mechanism by which RASSF1A modulates the formation of TNFRC, we performed experiments to determine whether RASSF1A interacts with components of TNFRC. A previous report has shown the association of RASSF1A with TNFR1 in the U2OS osteosarcoma cell line.^20^ However, it is not known whether RASSF1A binds to TRAF2 and TRADD and whether it links these molecules to TNFR1 following TNF-α stimulation. We conducted co-immunoprecipitation experiments in isolated neonatal rat cardiomyocytes to examine the possible protein–protein interactions between different components of the TNFRC (TNFR1, TRAF2, and TRADD) and RASSF1A in the presence of 10 ng/mL of TNF-α. Results shown in *Figure 3C* revealed that endogenous RASSF1A co-precipitated with TNFR1, TRAF2, and TRADD in cardiomyocytes in the presence of TNF-α. These data strongly suggested that upon TNF-α stimulation RASSF1A was recruited to the TNFRC, where it might play an important role as an adapter molecule which links TRAF2 and TRADD to TNFR1.

TNF-α receptor 2 (TNFR2) has been described to have a protective role in the heart.^21^ Therefore, it is important to examine whether TNFR2 is also involved in the RASSF1A-dependent TNF-α regulation. However, immunoprecipitation analysis showed that RASSF1A did not interact with TNFR2 (*Figure 3C*). This suggests that the RASSF1A regulatory mechanism was unlikely via TNFR2.

### 3.5 RASSF1A interacts with TRAF2 and TRADD via its C-terminal end

To further define the mechanism by which RASSF1A modulates TRAF2 and TRADD recruitment to TNFR1, we performed co-immunoprecipitation analysis using deletion mutants of the RASSF1A protein as described previously.^15^ In brief, we have generated: (i) RASSF1A-ΔN, which has the N-terminal region (amino acids 1–132) deleted; (ii) RASSF1A-ΔC mutant, which lacks 53 amino acids at the carboxy terminal region, thereby removing the Salvador-RASSF-Hippo (SARAH) domain; and (iii) RASSF1A-ΔC + RA, which lacks both the RA (Ras binding) and SARAH domains (see Supplementary material online, *Figure S2*). We generated adenoviruses to enable expression of these mutant proteins in neonatal rat cardiomyocytes, which was confirmed by western blot (*Figure 3D*). Immunoprecipitation experiments showed that only the RASSF1A-ΔN co-precipitated with TRAF2 and TRADD. Both of the C-terminal deletion RASSF1A mutants did not bind to either TRAF2 or TRADD, indicating that the C-terminal region of RASSF1A is likely to be important in mediating interaction with both TRAF2 and TRADD (*Figure 3E* and *F*).

### 3.6 RASSF1A gene silencing in cardiomyocytes reduces TRAF2 and TRADD recruitment to TNFR

**Figure 4:**
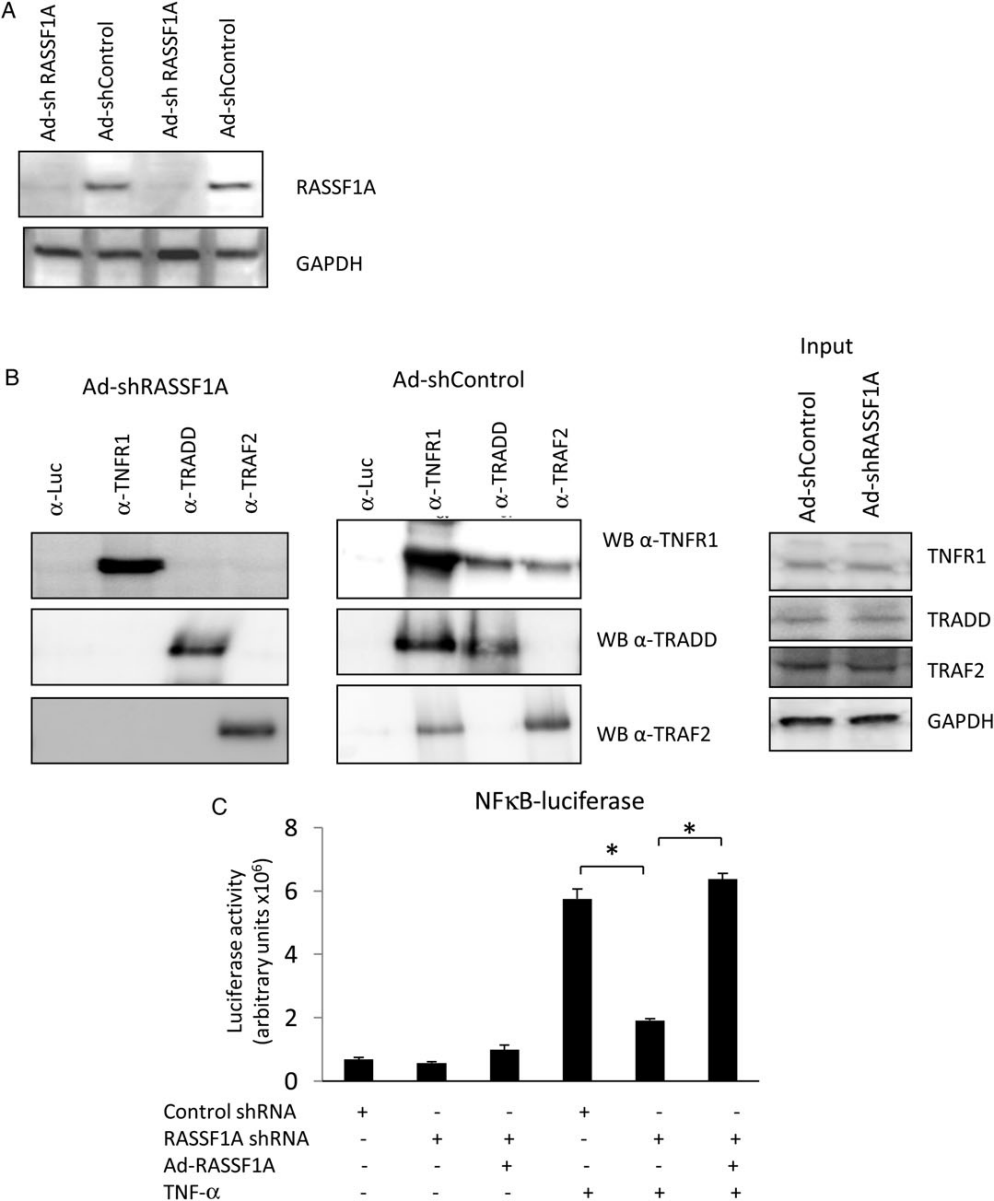
RASSF1A gene knockdown using shRNA reduces the formation of TNFRC and the activation of NFκB pathway. (*A*) Western blot analysis showed ablation of RASSF1A expression in NRCM treated with Ad-shRASSF1A. (*B*) Immunoprecipitation experiments suggested a significant reduction in the interaction between TNFR1–TRAF2 and TNFR1–TRADD in NRCM treated with Ad-shRASSF1A. All cells were stimulated with 10 ng/mL of TNF-α. (*C*) Activation of the NFκB pathway was assessed using adenoviral-driven NFκB-luciferase construct. RASSF1A gene inactivation significantly reduced NFκB activation in response to TNF-α induction. This phenotype was restored by overexpression of human RASSF1A (*P < 0.05, *n* = 3 independent experiments).

The phenotype described in *Figure 3A* and *B* was the result of systemic RASSF1A knockout in mice. To assess the effects of acute inactivation of RASSF1A, we performed a RNAi gene silencing approach. Adenovirus expressing RASSF1A shRNA was used to effectively knockdown RASSF1A expression in cardiomyocytes (*Figure 4A*). We then performed analyses to assess whether RASSF1A acute gene silencing altered the interaction between TNFR1 and TRAF2/TRADD. Co-immunoprecipitation experiments presented in *Figure 4B* showed that RASSF1A gene silencing markedly reduced TRAF2 and TRADD recruitment to TNFR1, demonstrating a consistent phenotype between systemic gene ablation in mice and acute gene silencing using the RNAi approach in isolated myocytes.

### 3.7 RASSF1A regulates NFκB activation in cardiomyocytes

TNF-α regulates several important signalling pathways in cardiomyocytes, such as the NFκB pathway, through binding to the TNFRs.^22^ To investigate whether alteration of RASSF1A expression modifies NF-κB signalling, we performed experiments using adenoviral-mediated NF-κB luciferase reporter. Using this system, we were able to detect activation of the NF-κB pathway in response to TNF-α in control cells. We found a significantly lower NF-κB luciferase activity in cardiomyocytes lacking RASSF1A following TNF-α treatment (*Figure 4C*). Importantly, overexpressing human RASSF1A in cells lacking this molecule rescued the NF-κB luciferase activation to the same level as control cells (*Figure 4C*). These data strongly support the notion of the important regulatory role of RASSF1A in TNF-α signalling.

### 3.8 RASSF1A is essential in regulating downstream TNF-α signalling

**Figure 5:**
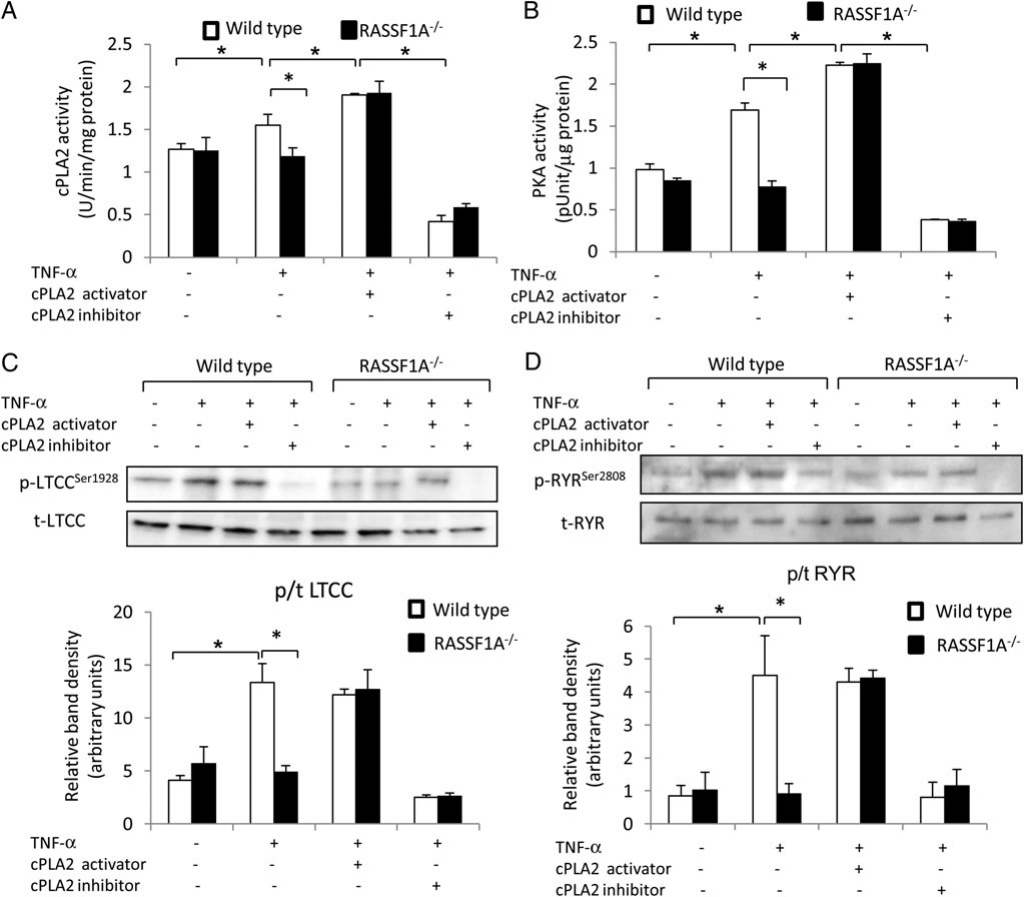
RASSF1A regulates cPLA2 activation and phosphorylation of calcium handling molecules. (*A*) cPLA2 activity was measured in isolated adult cardiomyocytes from RASSF1A^−/−^ mice and WT littermates treated with 10 ng/mL of TNF-α for 30 min. (*P < 0.05, *n* = 7–8) with or without the addition of cPLA2 activator peptide (PLAP; 1 μM) or cPLA2 inhibitor (AACOCF3; 20 μM). (*B*) PKA activity was significantly lower in RASSF1A^−/−^ cardiomyocytes following treatment with TNF-α (*P < 0.05, *n* = 7–8). Treatment with cPLA2 activator rescued the reduction of PKA activity in RASSF1A^−/−^ cardiomyocytes, whereas cPLA2 inhibitor reduced PKA activity of WT to the level similar with RASSF1A^−/−^. Western blot and band density measurement of (*C*) phosphorylated/total LTCC and (*D*) phosphorylated/total RYR showed a significant reduction in phosphorylation of LTCC and RYR in RASSF1A^−/−^ cardiomyocytes following TNF-α treatment. Treatment with cPLA inhibitor AACOCF3 (20 mM) abolished LTCC and RYR phosphorylation in WT cells to the level comparable with RASSF1A^−/−^ myocytes, whereas cPLA2 activator (PLAP; dose) rescued the phenotype of RASSF1A^−/−^ cardiomyocytes (*P < 0.05, *n* = 6–8).

Two possible downstream effectors of the TNF-α signalling pathway were investigated to gain mechanistic insights into the regulation of cardiomyocyte calcium dynamics, i.e. the cPLA2 and the CaMKII. We isolated adult cardiomyocytes from RASSF1A^−/−^ and WT mice and treated them with 10 ng/mL of TNF-α for 30 min. Measurement of cPLA2 and CaMKII activities showed that the activities of both enzymes were significantly increased in response to TNF-α treatment; however, only cPLA2 activity was markedly reduced in RASSF1A-deficient cardiomyocytes (*Figure 5A* and Supplementary material online, *Figure S3*), suggesting that cPLA2 was likely the downstream effector of RASSF1A signalling. In cardiomyocytes, cPLA2 activation will enhance the formation of arachidonic acid (AA),^7^ which may eventually modulate intracellular calcium via a variety of systems, including ion channel activation and induction of protein kinase A (PKA) activity.^23,24^ Consistently, PKA activity was significantly reduced in RASSF1A^−/−^ cardiomyocytes following TNF-α induction (*Figure 5B*).

Next, we analysed activation of calcium handling proteins that are essential in regulating calcium transient amplitude. We focused on measuring the phosphorylation levels of L-type calcium channel (LTCC) and ryanodine receptor (RYR). Western blot analysis showed that TNF-α stimulation significantly increased the phosphorylation of LTCC and RYR in WT cardiomyocytes; however, this effect was completely ablated in RASSF1A^−/−^ cardiomyocytes (*Figure 5C* and *D*).

**Figure 6:**
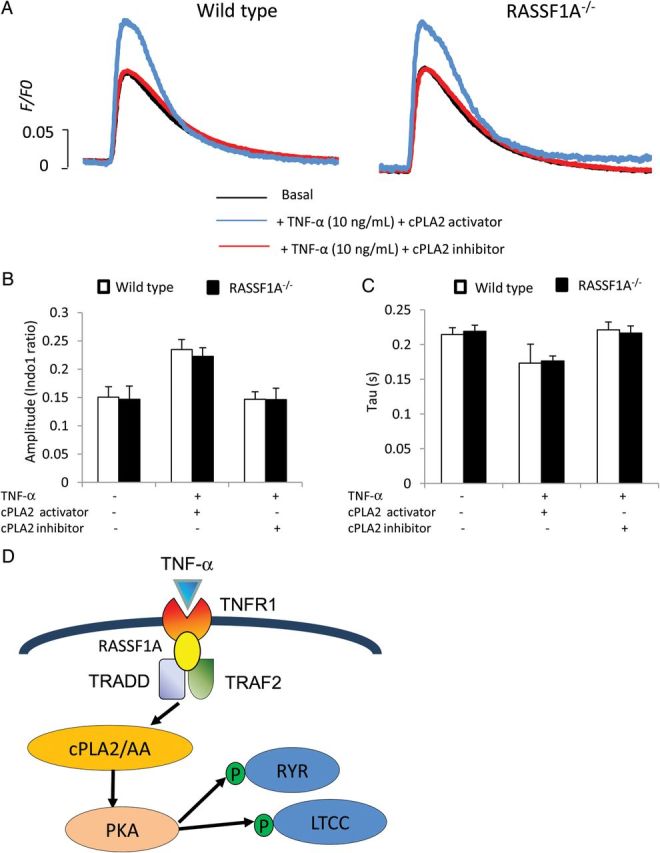
Effects of cPLA2 inhibition and activation on TNF-α induced calcium transients. (*A*) Representative calcium transient traces from WT and RASSF1A^−/−^ adult cardiomyocytes loaded with Indo-1 dye at basal and after stimulation with TNF-α (10 ng/mL) for 30 min in the presence of cPLA2 activator peptide (PLAP; 1 μM ,blue line) or cPLA2 inhibitor (AACOCF3, 20 μM, red line). (*B*) Analysis of calcium transient amplitude and (*C*) time constant of calcium decay (Tau) indicated that inhibition of cPLA2 abolished the TNF-α-induced elevation of calcium transient in WT cardiomyocytes, whereas cPLA2 rescued the reduction of TNF-α-induced contractile response in RASSF1A^−/−^ myocytes as shown in *Figure 2* (*n* = 25–30 cells in each group taken from four animals). (*D*) Schematic diagram of possible downstream signalling pathway regulated by RASSF1A.

To further ascertain if RASSF1A regulates TNF-α-dependent calcium transients via cPLA2, we performed analysis using cPLA2 inhibitor (AACOCF3, 20 µM) as well as cPLA2 activator peptide (PLAP, 1 μM). Analysis of calcium transients in WT and RASSF1A^−/−^ cardiomyocytes revealed that treatment with AACOCF3 reduced the TNF-α-induced elevation of Ca^2+^ transient amplitude in WT myocytes to a level comparable with that produced by RASSF1A^−/−^ myocytes (*Figure 6A* and *B*). No further effect of cPLA2 inhibition was observed in RASSF1A^−/−^ myocytes. On the other hand, cPLA2 activator was able to rescue the reduction of TNF-α-induced elevation of Ca^2+^ transient amplitude in RASSF1A^−/−^ myocytes (*Figure 6A* and *B*). No difference was observed in calcium decay rate between WT and RASSF1A^−/−^ after cPLA2 inhibition or activation (*Figure 6C*). Moreover, inhibition of cPLA2 reduced phosphorylation of LTCC and RYR following TNF-α treatment in WT cardiomyocytes, whereas treatment with PLAP rescued the reduction in LTCC and RYR phosphorylation as well as the reduction in PKA activity in the RASSF1A^−/−^ cardiomyocytes (*Figure 5A–D*).

Taken together, our results suggest that the downstream mechanism by which RASSF1A affects TNF-α contractile response is likely through cPLA2 and subsequently the phosphorylation of calcium handling proteins such as RYR and LTCC; via activation of PKA (*Figure 6D*).

### 3.9 RASSF1A is involved in the regulation of lipopolysaccharide-induced contractile dysfunction

Endotoxemia is an important pathophysiological condition affecting cardiac contractile function. TNF-α is one of the key mediators of systemic endotoxemia.^10^ We therefore studied whether RASSF1A is involved in this process. To model systemic endotoxemia in mice, a single bolus of bacterial lipopolysaccharide (LPS, 20 mg/kg BW) was injected intraperitoneally. Cardiac function and serum TNF-α level were assessed 16 h after injection.

**Figure 7:**
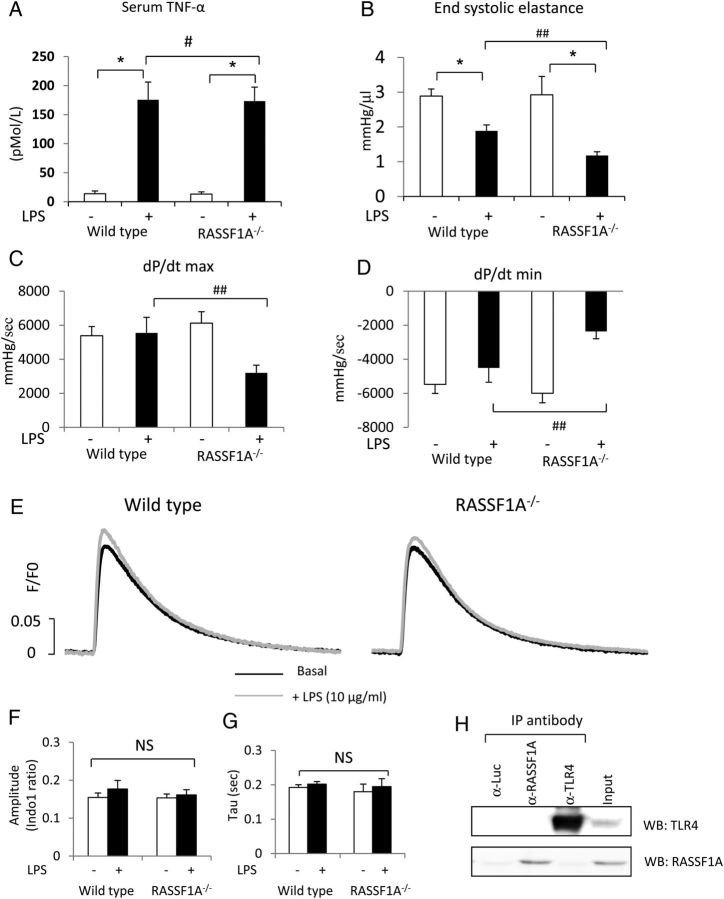
*In vivo* cardiac contractility following LPS infusion. (*A*) Serum TNF-α level was markedly elevated following treatment with LPS (20 mg/kg BW) for 16 h. ^#^Two-way ANOVAs indicated that there was a significant effect of LPS, but there was no significant interaction between genotype and TNF-α level. *Post hoc multiple comparison test showed that there was a significant difference in TNF-α levels between the LPS and non-LPS group (*P* < 0.05). (*B*) Analysis of Ees values, (*C*) d*P*/d*t*max, and (*D*) d*P*/d*t*min in mice after LPS treatment. *Two-way ANOVAs showed that there was a significant effect of LPS treatment and interaction between two factors tested (genotype vs. LPS). ^##^*Post hoc* multiple comparison showed that RASSF1A^−/−^ mice exhibited a significantly lower contractility compared with WT (*n* = 5–6 per group). (*E*) Representative calcium transients in response to LPS stimulation (10 μg/mL, 60 min) in WT and RASSF1A^−/−^ adult cardiomyocytes. (*F*) Quantification of calcium amplitude and (*G*) Tau. Two-way ANOVAs indicated that there was no significant interaction between genotype and treatment. (*F*) Immunoprecipitation analysis of isolated WT cardiomyocytes showed that RASSF1A did not interact with TLR4 in cardiomyocytes.

The levels of serum TNF-α were massively increased following LPS injection; however, we did not observe any difference between WT and RASSF1A^−/−^ mice (*Figure 7A*). Haemodynamic analysis showed that LPS-treated RASSF1A^−/−^ mice exhibited a significantly lower contractile function compared with LPS-treated WT mice as indicated by Ees, d*P*/d*t*max, and d*P*/d*t*min values (*Figure 7B–D*). We then treated isolated adult cardiomyocytes from WT and knockout mice with LPS (10 µg/mL) for 60 min and analysed the calcium transients (*Figure 7E–G*). We found that there was no significant difference regarding the change in calcium amplitude as well as the rate of calcium decay following direct LPS treatment between WT and RASSF1A^−/−^ cardiomyocytes. Furthermore, there was no difference in cPLA2 and PKA activities between WT and RASSF1A^−/−^ cardiomyocytes (see Supplementary material online, *Figure S4A* and *B*). Consistently, western blot analysis suggested that the levels of LTCC and RYR phosphorylation were not altered in RASSF1A^−/−^ cardiomyocytes following direct LPS treatment (see Supplementary material online, *Figure S4C* and *D*). We also performed immunoprecipitation analysis to assess whether RASSF1A interacts with LPS receptor, Toll-like receptor 4 (TLR4) in cardiomyocytes. We found that RASSF1A did not co-precipitate with TLR4 in the cardiomyocytes (*Figure 7H*). Taken together, our data suggest that the difference in the *in vivo* contractility was unlikely due to the direct LPS effect on cardiomyocytes.

## 4. Discussion

Our study shows that RASSF1A is an essential component for the formation of the TNFRC in cardiomyocytes and its subsequent downstream positive inotropic effects. We found that RASSF1A is a key adaptor molecule that facilitates the recruitment of TRAF2 and TRADD to the TNFR1 upon TNF-α stimulation, and this process is important in the regulation of downstream signal transmission. In the absence of RASSF1A, little if any TRAF2 and TRADD are recruited to the TNFRC. Functionally, the absence of RASSF1A virtually ablates the contractile response to TNF-α. Importantly, the β-adrenergic inotropic response was not altered in RASSF1A^−/−^ cardiomyocytes, suggesting that the effect of RASSF1A ablation seems to be specific to TNF-α signalling.

Previous reports have shown that acute application of low doses of TNF-α produces an increase in calcium transient amplitude and cardiac contractility.^8,9^ In RASSF1A^−/−^ mice and adult cardiomyocytes isolated from RASSF1A^−/−^, these effects were abolished. We proposed that RASSF1A facilitates the association of TRAF2 and TRADD, two molecules essential for TNF signalling, with the TNFRC. In the absence of RASSF1A, less TRAF2 and TRADD were recruited to the complex upon stimulation with TNF-α, leading to a reduction in downstream phospholipase-mediated signal transmission and hence a reduction in the calcium amplitude and contractility. Previously, it has been described that the N-terminus of RASSF1A is responsible for the interaction with TNFR1^20^ in the U2OS osteosarcoma cell line. In this study, we show the interaction of RASSF1A with TRAF2 and TRADD, which is likely mediated by the C-terminal region of RASSF1A. Therefore, we speculate that, in the process of TNFRC formation, RASSF1A may act as an adaptor molecule linking TRAF2/TRADD (via its C-terminal domain) to TNFR1 (via its N-terminal domain).

In various cell types, including cardiomyocytes, TNF-α stimulation induces cPLA2 activation.^25^ It has been shown previously that cPLA2 activation requires phosphorylation by p38 and caspase-mediated cleavage for its activation.^26,27^ TRAF2 is important in the TNFα-induced p38 phosphorylation via receptor-interacting protein and germinal centre kinase,^28^ whereas TRADD is crucial in the TNFα-induced caspase activation.^29^ Further downstream, active cPLA2 induces the release of AA from the cell membrane and hence elevation of AA level.^25^ AA has the capability to induce calcium entry from the intracellular calcium store.^23^ This process is dependent on PKA and anchoring protein for PKA (AKAP) activities, since inhibition of PKA^30^ and disruption of AKAP function^23^ abolish AA-induced calcium release. Taken together, these reports suggest that cPLA2 may activate PKA via AA and AKAP may play an important role in the spatial regulation of this signal.

Another possible downstream effector of TNF-α stimulation is CaMKII. Although as expected we observed significant elevation of CaMKII activity following TNF-α stimulation, we did not find any difference between WT and RASSF1A^−/−^ cardiomyocytes. This suggests that the TNF-α-induced CaMKII activation is unlikely to be mediated by the RASSF1A-dependent pathway. Indeed, it has been shown that the TNF-α-induced CaMKII activation is mostly mediated by oxidation and autophosphorylation processes due to the elevation of free radicals.^31,32^ This might explain our finding that the CaMKII activation was not different between WT and RASSF1A^−/−^ cardiomyocytes.

In this study, we found that ablation of RASSF1A led to the reduction in cPLA2 activity as well as decreased phosphorylation of major calcium handling proteins, such as LTCC and RYR, following TNF-α stimulation. In fact, cPLA2 inhibition using AACOCF3 abolished the TNF-α-induced inotropic response (elevation of intracellular Ca^2+^ amplitude) as well as the phosphorylation of LTCC and RYR in WT cardiomyocytes, whereas treatment with cPLA2 activator (PLAP) rescued the phenotype of RASSF1A^−/−^ myocytes. Taken together, our data present one possible mechanism explaining the reduction in contractile response and calcium transients in RASSF1A^−/−^ cardiomyocytes following TNF-α stimulation: ablation of RASSF1A alters the formation of the TNFRC, in particular the recruitment and activation of TRAF2 and TRADD, which subsequently causes a reduction in cPLA2 activation. This will result in the decreased activity of the downstream effectors, possibly PKA, which will eventually lead to the reduction in LTCC and RYR phosphorylation.

Previous publications have reported that RASSF1A acts as an inhibitor of NFκB signalling in fibroblasts^33^ and colon epithelium.^34^ This may suggest a cell-specific function of RASSF1A, since our observation suggests that, in cardiomyocytes, RASSF1A facilitates signal transmission from TNFR1 to the downstream targets including NFκB. It is also important to note that in the previous reports NFκB signal was assessed in unstimulated fibroblasts^33^ or in LPS-stimulated colon epithelium,^34^ whereas in the present observation we used TNF-α to stimulate cardiomyocytes.

While we found a strong association between RASSF1A, TNFR1, and its molecular complex, we did not observe interaction between RASSF1A and TNFR2. Although a previous publication has shown the involvement of TNFR2 in modulating cPLA2 activity,^35^ it is known that soluble TNF-α is inefficient in activating TNFR2,^36^ and hence treatment with soluble TNF-α will mainly activate TNFR1.^37^ This might be an explanation for our finding that RASSF1A^−/−^ displayed a completely attenuated response to TNF-α, since we treated cardiomyocytes with soluble TNF-α and hence there was no response of TNFR2. Another possible explanation is related to the signalling crosstalk between TNFR1 and TNFR2. It has been described that these receptors can influence each other's functions, for example, TNFR2 function might be dependent on TNFR1 since signalling via TNFR1 is required for the expression of several molecules which are important in mediating TNFR2 function, such as TRAF1 and cIAP1.^38^ It is possible therefore that, in RASSF1A^−/−^ myocytes, TNFR2 was indirectly inhibited as a result of reduced TNFR1 signalling. Indeed, further studies need to be performed to address the role of RASSF1A in TNFR2 signalling.

RASSF1A also forms complex with other membrane molecules, notably the plasma membrane calcium ATPase 4 (PMCA4).^39^ PMCA4 is a calcium extrusion pump which is located in the caveolae.^40^ Since TNFR1 is also enriched in the caveolae,^41^ it is possible that PMCA4 is involved in the regulation of TNFR1 signalling, although further studies need to be done to test this idea.

The present study also provides initial evidence of the possible involvement of RASSF1A in LPS-induced cardiomyopathy. In response to LPS injection, RASSF1A^−/−^ mice displayed a significant decrease in contractility compared with WTs. Interestingly, no differences in Ca^2+^ amplitude, cPLA2, and PKA activities or phosphorylation of LTCC and RYR were observed when we directly challenged isolated cardiomyocytes with LPS. Also, we found that RASSF1A did not interact with TLR4 in cardiomyocytes. Therefore, we speculated that this phenotype might be caused by the different response to TNF-α signalling. Both WT and RASSF1A^−/−^ mice produced comparable levels of TNF-α in the serum following LPS injection; however, since RASSF1A^−/−^ mice exhibited an attenuated inotropic response to TNF-α, this could account for the reduced contractility exhibited by RASSF1A^−/−^ mice following LPS injection (see Supplementary material online, *Figure S5*). It should be noted that further experiments need to be done to clarify the exact mechanism by which RASSF1A modulates LPS-induced cardiomyopathy. For example, a rescue experiment by activating the cPLA2–PKA pathway in RASSF1A^−/−^ mice during LPS stimulation is needed to elucidate whether this pathway is responsible in mediating the observed phenotype.

In summary, our present work demonstrates a novel TNF-α effector pathway mediated by RASSF1A which transmits the positive inotropic effect of TNF-α in cardiomyocytes. The complete understanding of the effector pathways of TNF-α is pivotal that may enable us to develop molecules that preserve the beneficial effect of TNF-α in the heart. This could be very useful for the treatment of conditions involving acute TNF-α elevation such as endotoxemia.

## Supplementary material

Supplementary material is available at *Cardiovascular Research* online.

**Conflict of interest:** none declared.

## Funding

This work was supported byBritish Heart Foundation (BHF) Intermediate Fellowship Grant (FS/09/046/28043) and BHF Project Grant (PG/11/23/28801) to D.O, and a Medical Research Council (MRC) Research Grant (G0802004) to L.N.

## Supplementary Material

Supplementary Data
