# Supplementary material for: The tumour suppressor Ras-association domain family protein 1A (RASSF1A) regulates TNF-α signalling in cardiomyocytes
Source: Cardiovasc Res. 2014 Apr 28;103(1):47–59. doi: 10.1093/cvr/cvu111 (PMC4207857; doi:10.1093/cvr/cvu111)
Supplement: Supplementary Data [file supp_103_1_47__index.html]

The Tumor Suppressor Ras-Association Domain Family Protein 1A (RASSF1A) Regulates TNF-α Signaling in Cardiomyocytes — The tumour suppressor Ras-association domain family protein 1A (RASSF1A) regulates TNF-α signalling in cardiomyocytes — The tumour suppressor Ras-association domain family protein 1A (RASSF1A) regulates TNF-α signalling in cardiomyocytes — Supplementary Data 

# The tumour suppressor Ras-association domain family protein 1A (RASSF1A) regulates TNF-α signalling in cardiomyocytes

## Supplementary Data

Supplementary Data

**Files in this Data Supplement:**

- Supplementary Data - Doc file
- Supplementary Figures - ppt file
