# Supplementary material for: The tumour suppressor Ras-association domain family protein 1A (RASSF1A) regulates TNF-α signalling in cardiomyocytes
Source: Cardiovasc Res. 2014 Apr 28;103(1):47–59. doi: 10.1093/cvr/cvu111 (PMC4207857; doi:10.1093/cvr/cvu111)
Supplement: Supplementary Data [file supp_cvu111_cvu111supp.doc]

**SUPPLEMENTARY METHODS**

**Adult cardiomyocyte isolation**

Adult cardiomyocytes were isolated from 3-4 month old animals. In brief, mice were sacrificed by cervical dislocation, the hearts were rapidly removed and then perfused via the aorta with isolation solution pH 7.34 (134mM NaCl; 11mM glucose; 4mM KCl; 1.2mM MgSO_4_; 1.2mM NaH_2_PO_4_; 10mM HEPES) for 4 minutes followed by 9 minutes perfusion with a solution containing 0.6mg/ml and 0.075mg/ml of collagenase type II (Worthington) and proteases type XIV (Sigma-Aldrich), respectively. Hearts were then perfused for 12 minutes with Tyrode solution containing 50mM taurine pH 7.34. The ventricles were cut from the heart and placed in Tyrode-taurine solution. The ventricles were then cut in half and pipetted up and down through a Pasteur pipette in 5 ml of Tyrode-taurine solution to release the cardiomyocytes.

**Intracellular calcium transient measurements**

Isolated adult cardiomyocytes were loaded with 5μM of the lipophilic form of Indo-l (Indo-l/AM, dissolved in dimethylsulfoxide) plus 5μM Pluronic F127 for 15 minutes in the dark at 37°C. The cells were then washed with Tyrode’s solution pH 7.4 (131mM NaCl, 4mM KCl, 1mM CaCl_2_, 1mM MgCl_2_, 10mM glucose and 10mM HEPES) and incubated for 20 min at 37°C to obtain complete de-esterification of the dye. In order to measure the cytosolic calcium the coverslips containing loaded myocytes were placed in a bath with a cover slip base. The bath was positioned on the stage of an epifluorescence adapted inverted Olympus IX70 microscope fitted with an Olympus America camera. The myocytes were perfused with Tyrode solution and then field stimulated by two silver wire electrodes initiating an electrical current at a frequency of 1Hz, calcium changes during myocyte contraction were recorded under basal conditions at 33^0^C. Excitation was set at 340 nm using an ARC lamp (Cairn Inc), by means of a laser spot applied to small areas of the cell. The fluorescence emission of both the free (485nm) and bound calcium (405nm) forms of the dye were collected using a dichromic filter and two Cairn Integra photomultiplier tubes. The intracellular calcium concentration was related to the changes in the ratio of the fluorescence signals (R = F_405_/F_485_). The data were collected and analysed using Ionwizard software (Ionoptix Inc.). Calcium was calibrated as previously described^1^. Briefly, Indo-1 ratio was converted into calcium concentration in nM using the equation: [Ca^2+^]_i_ in nM =K_d_ * β * [(R-R_min_)/(R_max_-R)], K_d_ = 250nM, β = ratio of 485-nm signal in low Ca^2+^ to that in high Ca^2+^, R is the Indo-1 ratio recorded by the cell in the presence of 1mM calcium, R_min_ is the Indo-1 ratio in presence of 10mM EGTA and 2μM ionomycin, R_max_ is the indo-1 ratio in the presence of 5 mM calcium chloride and 2μM ionomycin.

**Neonatal rat cardiomyocyte isolation**

We isolated neonatal rat cardiomyocyte (NRCM) from 1-3 day old Sprague Dawley rat neonates. Rat neonates were sacrificed by cervical dislocation followed by decapitation. Hearts were cut into small pieces and ventricular tissues were digested by several rounds of 7 minutes incubation in ADS buffer (0.68% NaCl (w/v), 0.476% Hepes (w/v), 0.012% NaH_2_PO_4_ (w/v), 0.1% glucose (w/v), 0.04% KCl (w/v), 0.01% MgSO_4_ (w/v), pH 7.35) containing 0.3 mg/ml collagenase A (Roche) and 0.6 mg/ml pancreatin (Sigma-Aldrich) at 37 °C with shaking. Digestions were pooled and cells were centrifuged and resuspended in plating media (68% DMEM, 17% Medium 199, 10% horse serum, 5% FCS). Cells were then plated on tissue culture dishes, and incubated for 60 min at 37 °C, 5% CO_2_ to separate cardiac fibroblasts. Supernatants containing cardiomyocytes were plated on collagen-coated tissue culture dishes and further incubated overnight at 37 °C, 5% CO_2_ to allow cellular attachment. The following day, cells were washed with PBS and plating media was replaced with maintenance media (79.5% DMEM, 19.5% Medium 199, 1% FCS) supplemented with 1% penicillin/streptomycin.

**Co-immunoprecipitation and western blotting**

Animals were killed by cervical dislocation, hearts were homogenized in RIPA buffer (1 x PBS, 1% Igepal, 0.5% sodium deoxycholate, 0.1% SDS, 20μM PMSF, 500ng/ml leupeptin, 1μg/ml aprotium, 500ng/ml pepstatin) and protein content determined using a BCA protein assay reagent kit (Pierce). For immunoprecipitation experiments, protein lysates (1ml) were pre-cleared by incubation with 200μl of protein G-agarose beads (Roche) and 5μl of anti-luciferase polyclonal antibody (1mg/ml, Promega) at 4^o^C for 1 hour. Beads were removed by centrifugation at 1000xg and 500μl of pre-cleared extracts were incubated overnight at 4 C with the desired antibody or as a control, an anti-luciferase polyclonal serum (Promega) and 40μl of the beads. Western blotting was conducted by separating equal amounts of protein on 8% polyacrylamide gels and electroblotting onto nitrocellulose membrane using the recommended transfer buffer. Blots were probed with antibodies selective for RASSF1A (Anti- RASSF1A, Santa Cruz Inc.), TNFR1 (anti-TNFR1, Abcam), TNFR2 (anti-TNFR2, Abcam), TLR4 (anti-TLR4, Abcam), Flag tag (anti-Flag, Sigma-Aldrich), TRADD (anti-TRADD, Abcam), TRAF2 (anti-TRAF2, Abcam), L-type calcium channels (anti-LTCC, Abcam), ser1928 L-type calcium channel (anti P-LTCC^ser1928^, Badrilla), RYR (anti-RYR, Abcam), ser2808phospho RYR (anti-RYR^ser2808^, Badrilla), and anti-mouse HRP (Dako) or anti-rabbit HRP (Jackson) were used as secondary antibodies. Immunocomplexes were visualized using enhanced chemiluminescence according to the manufacturer’s protocol (Amersham) and exposed to autoradiographic film (Kodak).

**NFκB luciferase assay**

7x10^5^ NRCM in 1ml maintenance media were plated per well of a 24 well BD Primaria tissue culture plates for 24 hours. The next day the media was changed and the cells were infected with 25 MOI of NFκB luciferase virus in addition to 25 MOI of the desired expressing adenovirus. NRCM were incubated for 72 hours to allow expression or knockdown of proteins. On the day of performing the luciferase assay, the cells were stimulated with TNF-α 10ng/ml for 30 minutes then the medium was removed and NRCM were washed twice with PBS. NRCM were lysed in cell culture lysis buffer (Promega Inc.) for 20 minutes at room temperature. Then 10μl of the cell lysate was placed in a luminometer tube. The luminometer (Berthold technologies Lumat LB 9507), was programmed to dispense 100μl of the luciferase substrate (luciferen) (Promega Inc.) in luciferase lysis buffer and to perform a two second measurement followed by two second measurement delay.

**cPLA2 activity assay**

Cytoplasmic phospholipase A2 (cPLA2) activity was assessed using cPLA2 assay Kit (Cayman) following the manufacturer’s protocol. Briefly, hearts were homogenized in a suitable amount of cPLA2 extraction buffer (50mM HEPES, pH 7.4, containing 1mM EDTA). To avoid any measurement of iPLA2 or sPLA2 the samples were pre-incubated with 10µM of bromoenol-lactone and thioetheramide-PC (specific inhibitors for iPLA2 and sPLA2) for 30 minutes. Then the tissue homogenate was incubated with Arachidonoyl Thio-PC as a substrate for cPLA_2_. Hydrolysis of the arachidonoyl thioester bond at the sn-2 position by PLA_2_ releases a free thiol which can be detected by DTNB (5,5'-dithiobis(2-nitrobenzoic acid)). The levels of free thiol released were determined by colorimetric measurement at 405nm wavelength. The readings were normalised to the protein content and converted to cPLA2 activity using a conversion factor provided by the kit manufacturer.

**PKA activity assay**

PKA activity was determined in isolated adult cardiomyocytes before or after TNF-α treatment as we described previously[{Mohamed, 2011 #535}](#_ENREF_4) using a PepTag® Assay for Non-Radioactive Detection of cAMP-Dependent Protein Kinase kit (Promega) following the manufacturer’s protocol. Briefly, isolated adult cardiomyocytes were homogenized in a suitable amount of PKA extraction buffer (25mM Tris-HCl pH 7.4, 0.5mM EDTA, 0.5mM EGTA, 10mM β-mercaptoethanol, 1μg/ml leupeptin, 1μg/ml aprotinin and 0.5mM PMSF). 10μl of cells were incubated with 2μg PepTag® A1 Peptide (L-R-R-A-S-L-G (Kemptide) for 30 minutes. The reaction product was run on a 0.8% agarose gel for 20 minutes and the phosphorylated peptide bands were isolated. The level of phosphorylation was determined by colorimetric measurement at 570nm wavelength. The readings were normalized to the protein content of each sample and converted to PKA activity unit using a standard curve of PKA enzyme.

**CAMKII activity assay**

We assessed the CAMKII activity in isolated adult cardiomyocytes before or after treatment with TNF-α using CAMKII Kinase Enzyme assay kit (Promega, Inc.) following the manufacturer’s protocol. Briefly, the cells where homogenized in a suitable volume of extraction buffer (25mM Tris-HCl pH 7.4, 0.5mM EDTA, 0.5mM EGTA, 10mM β-mercaptoethanol, 1μg/ml leupeptin, 1μg/ml aprotinin and 0.5mM PMSF). Then the 2X reaction buffer which contains 25mM MOPS pH 7.2, 12.5mM β-glycerol-phosphate, 25mM MgC12, 5mM EGTA, 2mM EDTA and 0.25mM DTT was prepared. The enzymatic reaction was performed in clear bottom 96-well plate, by adding the following reaction components bringing the initial reaction volume up to 25μl: 5μl of the cellular extract, 5μl of 1mg/ml stock solution of peptide substrate, 2.5μl of Ca2+/Calmodulin solution (10X), 7.5μl of 2X reaction Buffer. For each sample, a negative control was performed by adding 10µM KN-93 (known inhibitor for CAMKII) which was considered as a background reading. The CAMKII reactions were initiated by the addition of 5μl of 250μM ATP. The plates were incubated at 30 ^0^C for 30 minutes. The reaction was terminated by adding 25μl of ADP-Glo Reagent. The 96-well plate was agitated and the reaction incubated for 40 minutes at ambient temperature. 50μl of the Kinase Detection Reagent was added and then incubated for 30 minutes at ambient temperature. The luminescence activity was read in a 96 well plate Luminometer (BMG Labtach).

**REFERENCE**

1. Lagadic-Gossmann D, Buckler KJ, Le Prigent K, Feuvray D. Altered Ca2+ handling in ventricular myocytes isolated from diabetic rats. *Am J Physiol* 1996;**270**:H1529-1537.
