# Supplementary material for: The tumour suppressor Ras-association domain family protein 1A (RASSF1A) regulates TNF-α signalling in cardiomyocytes
Source: Cardiovasc Res. 2014 Apr 28;103(1):47–59. doi: 10.1093/cvr/cvu111 (PMC4207857; doi:10.1093/cvr/cvu111)
Supplement: Supplementary Data [file supp_cvu111_cvu111supp_figs.ppt]

## Slide 1
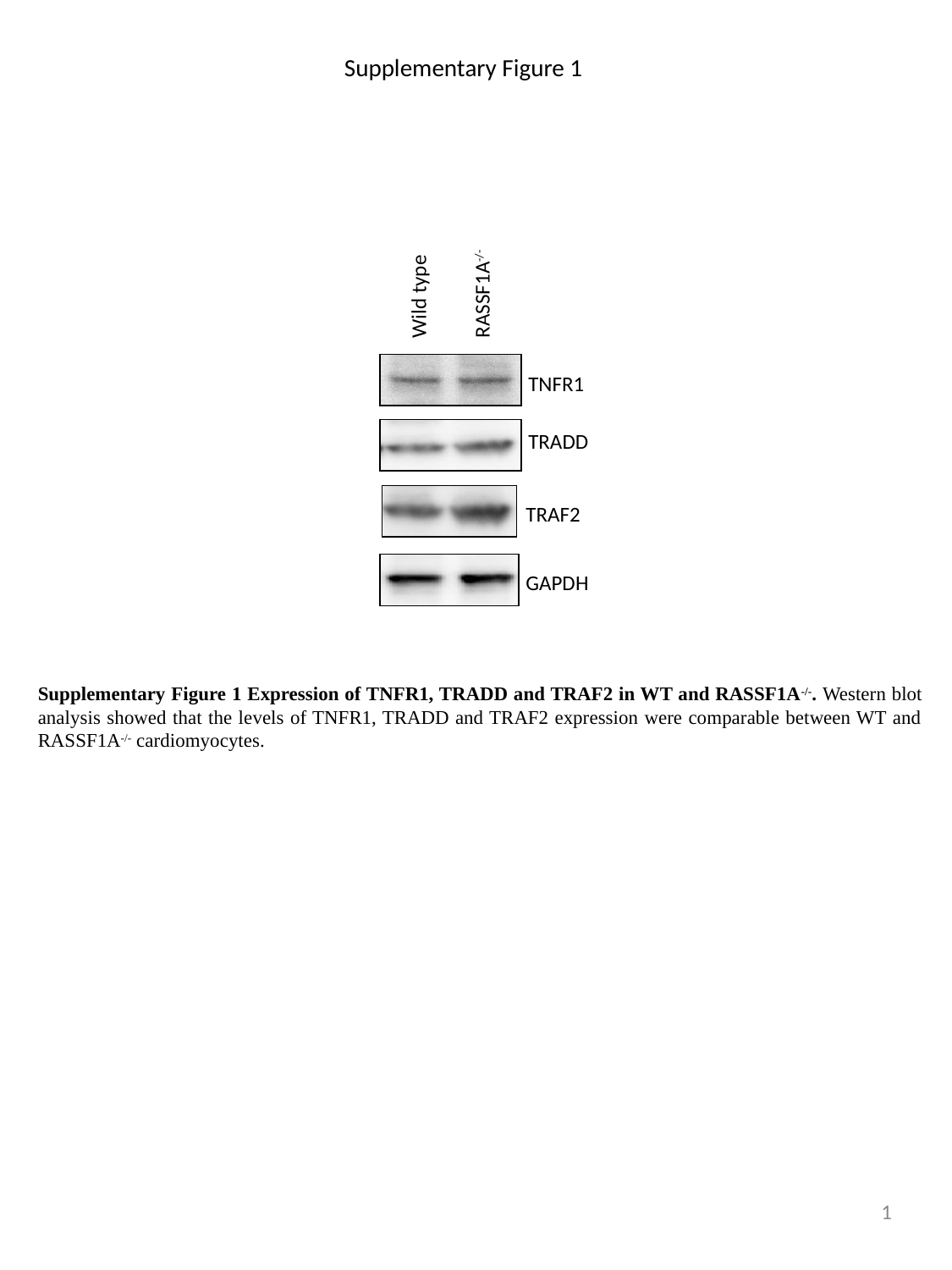

Supplementary Figure 1
Wild type
RASSF1A-/-
TNFR1
TRADD
TRAF2
GAPDH
Supplementary Figure 1 Expression of TNFR1, TRADD and TRAF2 in WT and RASSF1A-/-. Western blot analysis showed that the levels of TNFR1, TRADD and TRAF2 expression were comparable between WT and RASSF1A-/- cardiomyocytes.
1

## Slide 2
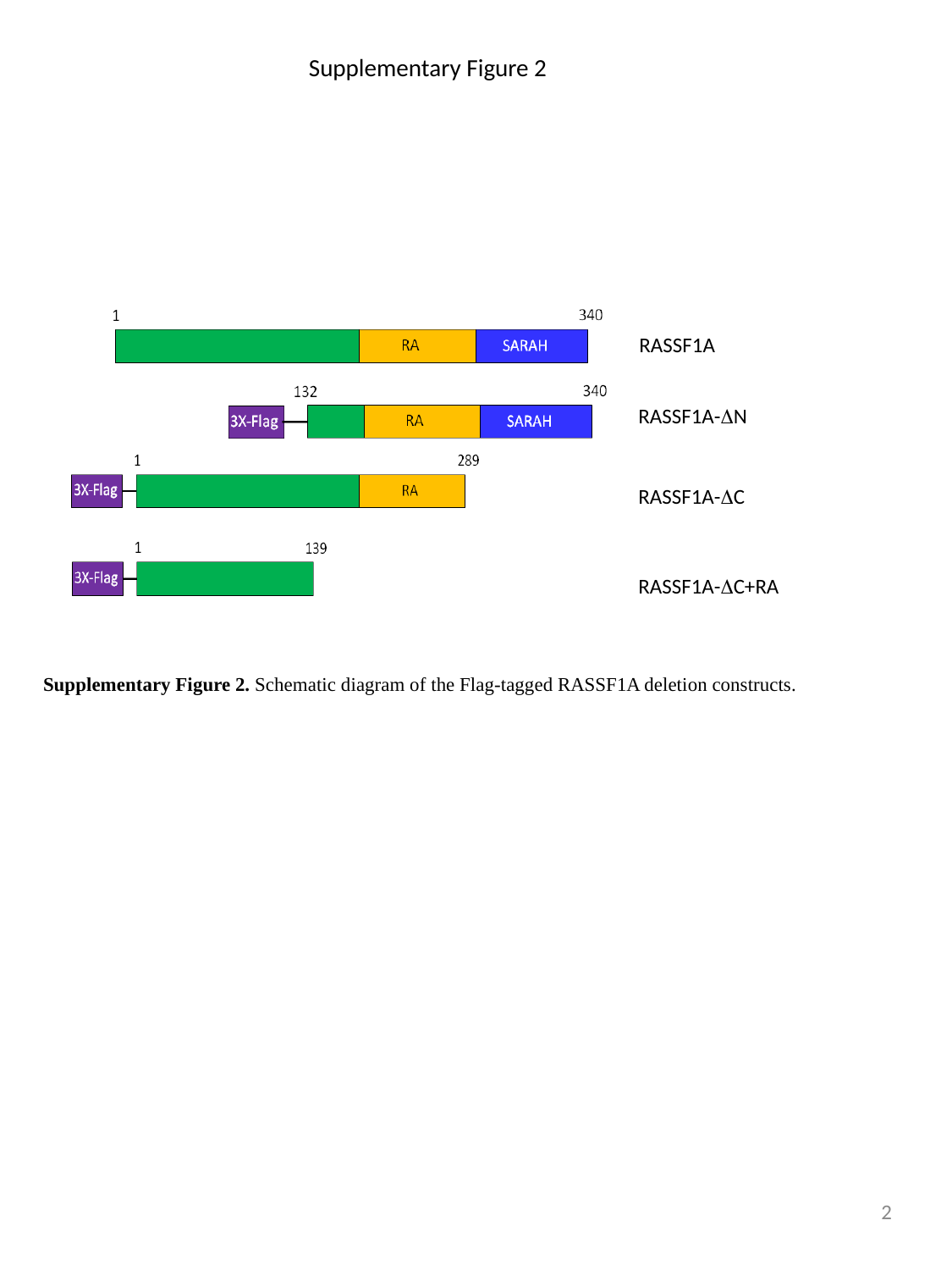

Supplementary Figure 2
RASSF1A
RASSF1A-ΔN
RASSF1A-ΔC
RASSF1A-ΔC+RA
Supplementary Figure 2. Schematic diagram of the Flag-tagged RASSF1A deletion constructs.
2

## Slide 3
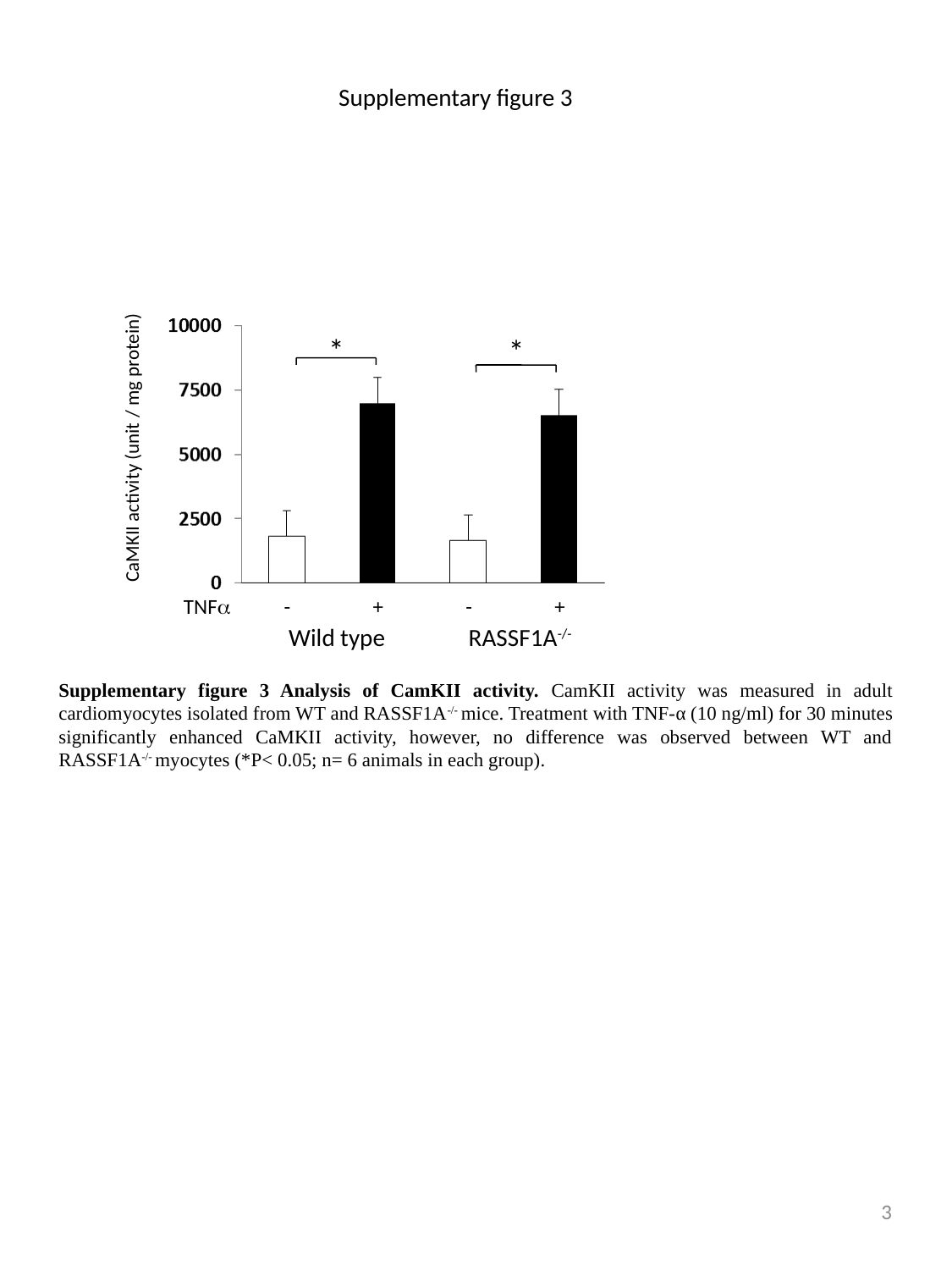

Supplementary figure 3
*
*
CaMKII activity (unit / mg protein)
TNFα - + - +
Wild type
RASSF1A-/-
Supplementary figure 3 Analysis of CamKII activity. CamKII activity was measured in adult cardiomyocytes isolated from WT and RASSF1A-/- mice. Treatment with TNF-α (10 ng/ml) for 30 minutes significantly enhanced CaMKII activity, however, no difference was observed between WT and RASSF1A-/- myocytes (*P< 0.05; n= 6 animals in each group).
3

## Slide 4
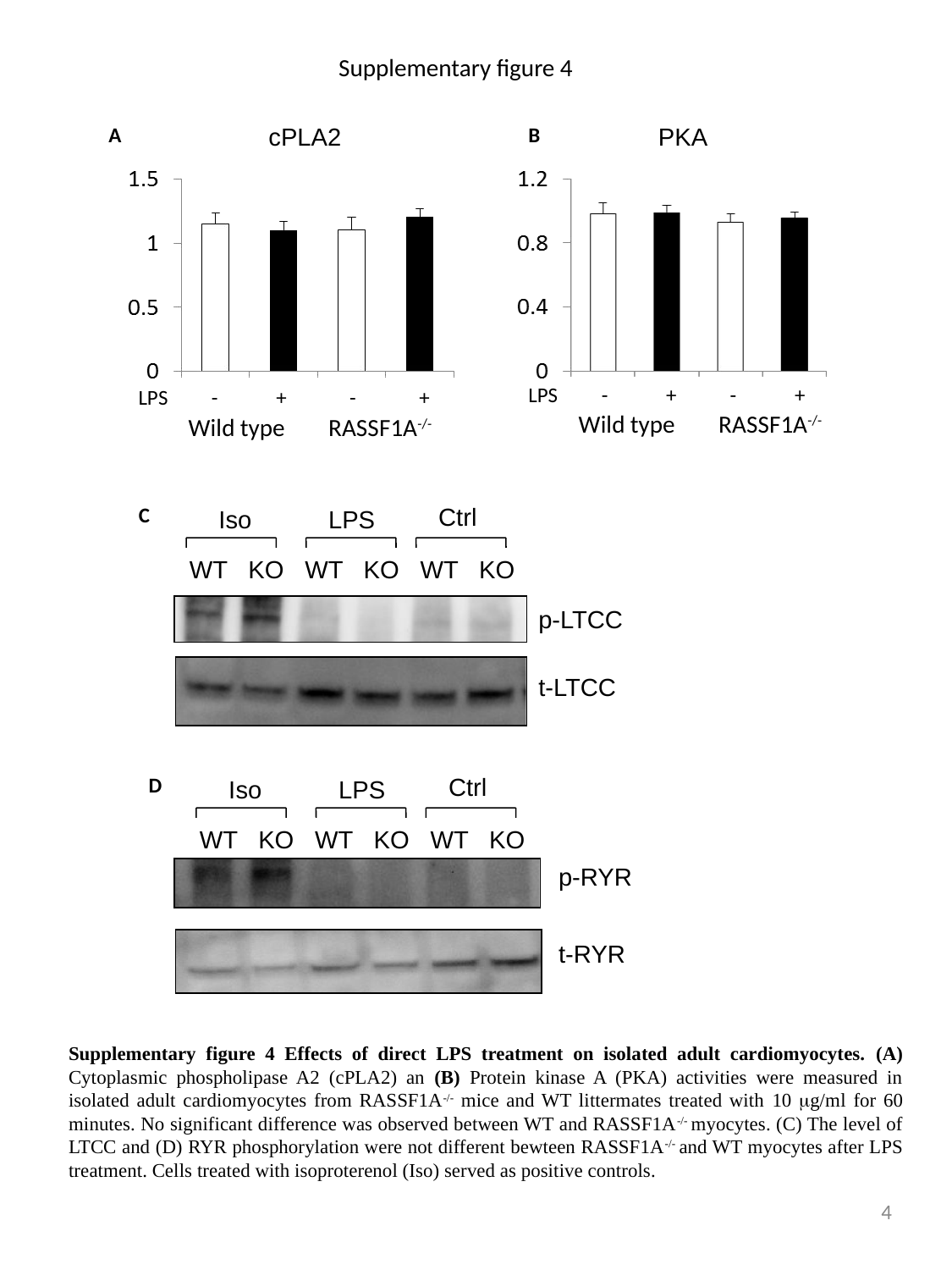

Supplementary figure 4
A
cPLA2
B
PKA
LPS - + - +
LPS - + - +
Wild type
RASSF1A-/-
Wild type
RASSF1A-/-
C
Ctrl
Iso
LPS
 WT KO WT KO WT KO
p-LTCC
t-LTCC
D
Ctrl
Iso
LPS
 WT KO WT KO WT KO
p-RYR
t-RYR
Supplementary figure 4 Effects of direct LPS treatment on isolated adult cardiomyocytes. (A) Cytoplasmic phospholipase A2 (cPLA2) an (B) Protein kinase A (PKA) activities were measured in isolated adult cardiomyocytes from RASSF1A-/- mice and WT littermates treated with 10 μg/ml for 60 minutes. No significant difference was observed between WT and RASSF1A-/- myocytes. (C) The level of LTCC and (D) RYR phosphorylation were not different bewteen RASSF1A-/- and WT myocytes after LPS treatment. Cells treated with isoproterenol (Iso) served as positive controls.
4

## Slide 5
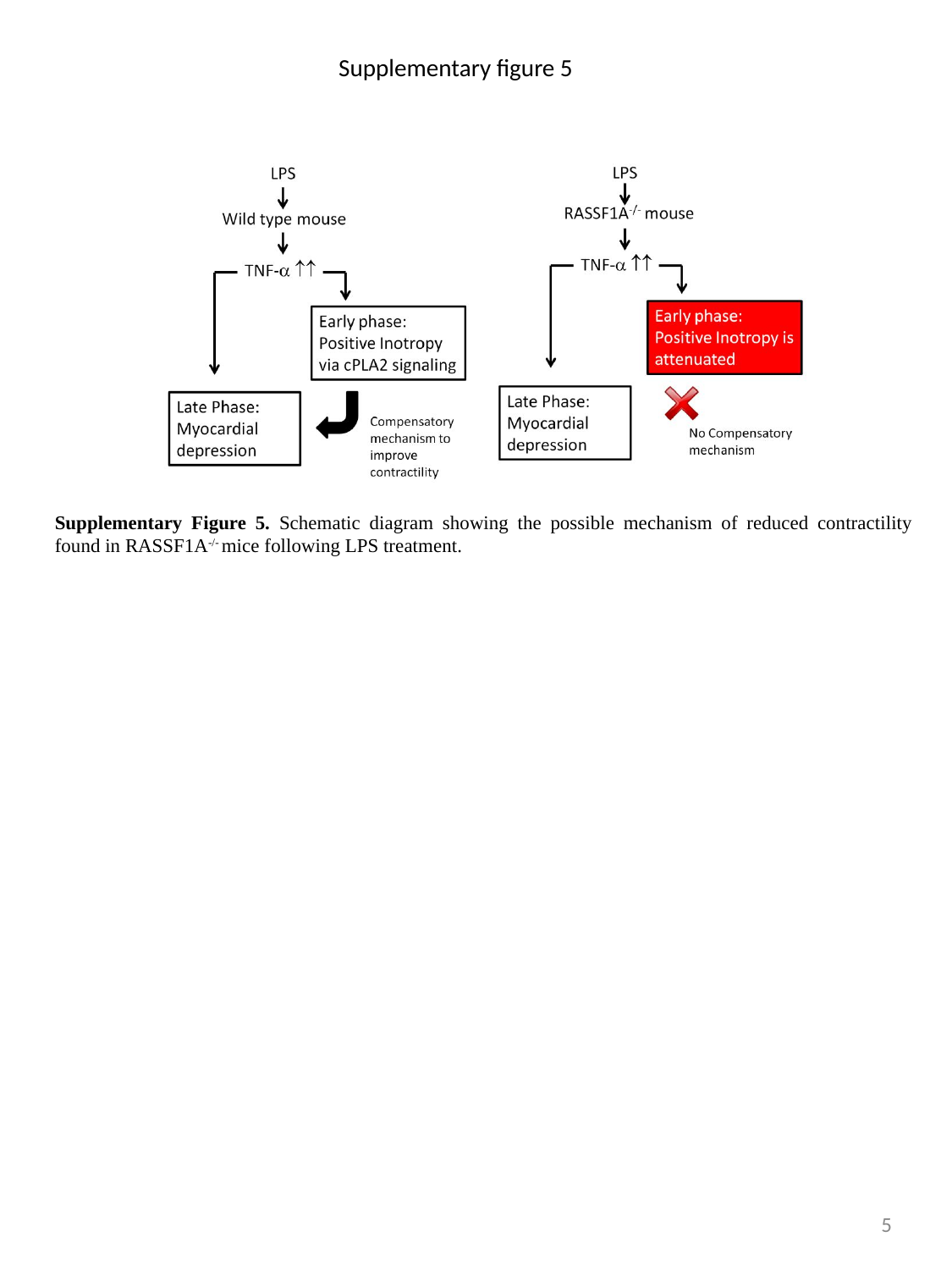

Supplementary figure 5
Supplementary Figure 5. Schematic diagram showing the possible mechanism of reduced contractility found in RASSF1A-/- mice following LPS treatment.
5
